# Development and Application of a Loop-Mediated Isothermal Amplification (LAMP) Approach for the Rapid Detection of *Dirofilaria repens* from Biological Samples

**DOI:** 10.1371/journal.pntd.0004789

**Published:** 2016-06-24

**Authors:** Donato Antonio Raele, Nicola Pugliese, Domenico Galante, Laura Maria Latorre, Maria Assunta Cafiero

**Affiliations:** Istituto Zooprofilattico Sperimentale della Puglia e della Basilicata, Unit of Medical Entomology, Department of Virology, Foggia, Italy; McGill University, CANADA

## Abstract

Dirofilariasis by *Dirofilaria repens* is an important mosquito vector borne parasitosis, and the dog represents the natural host and reservoir of the parasite. This filarial nematode can also induce disease in humans, and in the last decades an increasing number of cases have been being reported. The present study describes the first loop mediated isothermal amplification (LAMP) assay to detect *D*. *repens* DNA in blood and mosquitoes. Two versions of the technique have been developed and described: in the first, the amplification is followed point by point through a real time PCR instrument (ReT-LAMP); in the second, the amplification is visualized by checking UV fluorescence of the reaction mixture after addition of propidium iodide (PI-LAMP). The two variants use the same set of 4 primers targeting the *D*. *repens* cytochrome oxidase subunit I (COI) gene. To assess the specificity of the method, reactions were carried out by using DNA from the major zoonotic parasites of the family of Onchocercidae, and no amplification was observed. The lower limit of detection of the ReT-LAMP assay was 0.15 fg/μl (corresponding to about 50 copy of COI gene per μl). Results suggest that the described assay is specific, and its sensitivity is higher than the conventional PCR based on the same gene. It is also provide a rapid and cost-effective molecular detection of *D*. *repens*, mainly when PI-LAMP is applied, and it should be performed in areas where this emerging parasitosis is endemic.

## Author Summary

*Dirofilaria repens* is a filarial nematode which mainly infests the dog, but humans may be occasionally infested, too. The spread of the parasite is mediated by a number of mosquitoes species, which are well recognized as vectors of *D*. *repens*. The majority of reports of the disease come from the European Countries, especially those along the Mediterranean basin, but in the last decade several cases have been recorded also from Asian and African Countries, and this led the scientific community to consider such parasitosis an emerging disease. To date, diagnosis is based the morphologic analysis of microfilariae, isolated from the blood of infected hosts, but this may be time-consuming and the identification of parasite requires specialized parasitologists. The here described approach, based on the loop-mediated isothermal amplification (LAMP), allows the detection of *D*. *repens* genomic DNA directly from the biological samples, and it may be easily and rapidly performed, producing unequivocal results in less than a hour. We also presented two versions of the assays. The first, a real-time LAMP, is characterized by a very high sensitivity but it requires an expensive real time PCR instrument, while the second, performed with the addition of propidium iodide, does not need such equipment, therefore being very affordable. This makes it suitable to be carried out in field and whenever expensive equipment or specialized personnel lacks.

## Introduction

Dirofilariases are parasitic diseases caused by nematodes of the genus *Dirofilaria* (Nematoda: Onchocercidae) and transmitted by hemathophagous arthropods. The subgenera *Dirofilaria (Nochtiella)* is represented by 22 species including *Dirofilaria repens* Railliet et Henry, 1911 [1]. The latter is a common mosquito–borne parasite of subcutaneous tissues of dogs and others carnivores in the Old World [2], including wolf and fox [3]. Cats and feline in general are less susceptible to the infestation, which consists of a low microfilaremia [4–5]. *D*. *repens* has zoonotic potential: it can infest humans, and it is considered one of the most important vector-borne parasitosis in humans in Europe [6].

Among these hosts, the dog is the most important and it also act as a reservoir for the parasite [7–8]. From dog, the parasite may be transmitted to several species of mosquitoes (Diptera, Culicidae) [9], which have been proved to act as a *D*. *repens* vector [10–11]. Specifically, they may transmit infective third-stage larvae from animals with microfilariemia to humans during the blood-feeding. Therefore, although the nematode is not highly pathogenic for the dog, where it usually causes subcutaneous tissue diseases, it is considered of primary veterinary importance due to its zoonotic behavior [10].

In humans the parasite may locate itself in the subcutaneous tissues, mainly in the upper half of the body, but all regions may be potentially involved. Secondarily, it can migrate from the initial site of infection to others sites, commonly leading to subconjunctival and periorbital infestation [12], which may lead to severe ocular complications. Pulmonary forms have been reported, although rarely [13]. Microfilaremia has never been observed in humans, with only one exception [14].

The disease is widely diffused and, currently, the number of reports from humans and animals is increasing. Most of them come from the Mediterranean Basin, an endemic area of dirofilariasis, caused by both *D*. *repens* and *D*. *immitis* [10]. However, in the last years, cases were recorded not only from the endemic areas of Europe, including Italy, France Spain, Russia and Turkey [10, 15, 13, 16–17] but also from several Asiatic and African countries, such as India [18], Vietnam [19], Iran [20], Tunisia [21], Egypt [22], and South Africa [23]. This is leading the scientific community to consider the disease as an emerging zoonosis. Several factors are thought to contribute to this expansion, such as the increase in frequency and numbers of travels and movements of animals, the wider distribution of vector-competent mosquito species due to the international trade and global warming, and the improvement of the diagnostic techniques which enable more accurate detection of the pathogen [10, 15, 24].

These circumstances stress the need to have diagnostic tools effective in terms of accuracy, sensitivity and user-friendliness. A recently developed amplification technique, named loop-mediated isothermal amplification (LAMP), owns most of those features, so that it has been finding large application as a diagnostic tool [25]. A brief description of the principle and mechanism, as firstly described by Notomi et al. [26], is reported in the supplementary material S1 Text. This study describes two versions of a novel loop-mediated isothermal amplification assay for a rapid diagnosis of *D*. *repens* in dogs and mosquitoes.

## Material and Methods

### Primer design

**Fig 1:**
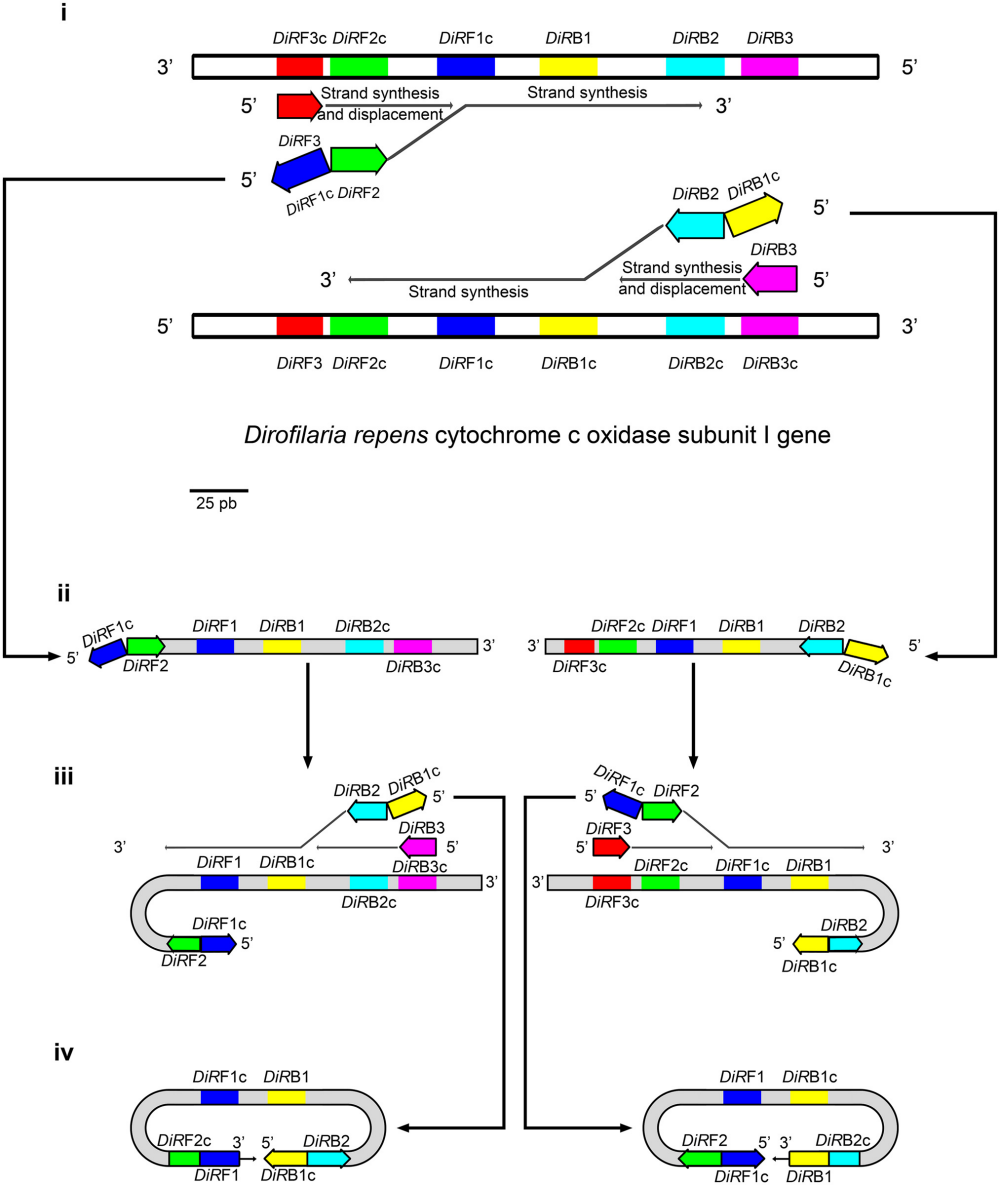
Relative positions of LAMP primers within the cytochrome c oxidase gene of *D*. *repens*. The first steps of the LAMP reaction are also depicted. i) Primers F3/B3, and *DIR*F2/*DIR*B2 (parts of *DIR*FIP and *DIR*BIP, respectively), recognize and anneal to their respective complementary targets. The DNA synthesis starts and the strand displacement activity of the DNA polymerase let the strand synthesized from *DIR*F2 and *DIR*B2 to be detached from the template strand. ii) The displaced neo-synthesized strands bring, at their termini, *DIR*F1c and *DIR*B1c that iii) will self-anneal to the *DIR*F1 and *DIR*B1 loci, respectively, thus forming a secondary structure with a loop. *DIR*B3/*DIR*F3 and *DIR*BIP/*DIR*FIP (specifically with the portions *DIR*B2 and *DIR*F2) hybridize with the complementary regions of the strand. The synthesis and the displacement of strand occur again, catalyzed by the same DNA polymerase. iv) the 3’ and 5’ termini, bearing *DIR*F1/*DIR*B1 and *DIR*B1c/*DIR*F1c, respectively, self-anneal to the complementary loci harbored by the same strand, which, in turn, will form the typical dumbbell structure. The DNA polymerase will catalyze the DNA synthesis starting from the 3’ terminus, displacing the 5’ terminus, and producing a concatamer which will be the basis for following the amplification steps.

The available sequences (Accession numbers AJ271614, AM749230, AM749231, AM749232, AM749233, AM749234, DQ358814, JF461458 and KF692102) of *D*. *repens* cytochrome oxidase subunit I (COI) from GenBank were aligned by ClustalW, implemented in MEGA 6.0 [27]. The resulting consensus sequence was then used to design LAMP primers by the mean of the Primer Explorer Program (Available online at http://primerexplorer.jp/e/, latest accessed 8^th^ April 2016). The primers are listed in Table 1. Because no complete sequences of *D*. *repens* COI are currently available, the relative positions of oligonucleotides are shown in Fig 1.

**Table 1:** List of primers for *D*. *repens* specific LAMP.

Primer name	Sequence 5’-3’
*DiR*FIP (*DiR*F1-*DiR*F2)	CCAGGTCCACCACCAATAAAAAAAG-TGTCTTTTTGGTTTACTTTTGTTGC
*DiR*BIP (*DiR*B1-*DiR*B2)	TCCTCCTTTAAGTGTTGATGGTCAA-CCAATACCTACAGTATGTAAACCCA
*DiR*F3	GCGTTTCCTCGTGTTAATGC
*DiR*B3	AACCATAAAATTAATAGCACCCAAC

### LAMP protocols

Two different variants of LAMP have been developed: a real-time LAMP (hereafter ReT-LAMP) and a propidium iodide LAMP (PI-LAMP). They differed for the visualization of results: in the first cases, the amplification is visualized as a curve in a real-time PCR instrument, while the second allows to visualize the amplification as UV fluorescence following the addition on propidium iodide.

The ReT-LAMP reactions were carried out in 25 μL containing 15 μL of Isothermal Master Mix with carboxyfluorescein (Optigene, Horsham, UK), primers *DiR*FIP and *DiR*BIP 0.4 μM each and primers *DiR*F3 and *DiR*B3 0.1 μM each. ROX was used as a passive reference dye. Five μL of total DNA were used as template. The mixture was incubated at 65°C for 40 min on a StepOne real time PCR system (Applied Biosystems, Milan, Italy).

The PI-LAMP reactions were carried out as previously described [28]. The reactions (final volume, 25 μL) were prepared by mixing the isothermal amplification buffer (20 mM Tris-HCl, 10 mM [NH_4_]_2_SO_4_, 50 mM KCl, 2 mM MgSO_4_, 0.1% Tween 20, pH 8.8), dNTPs 1.5 mM each, 1.6 μM each of the *DIR*FIP and *DIR*BIP primers, 0.3 μM each of the *DIR*F3 and *DIR*B3 primers, 2.5 μL of extracted DNA, and 8 U of *Bst* 2.0 Warm Start DNA Polymerase (New England Biolabs Inc., Ipswich, MA, USA). The reaction mixture was incubated in a heating block at 65°C for 45 minutes and then heated at 80°C for 5 minutes to terminate the reaction. Propidium iodide to a final concentration of 1 μg/μL was then added to the mixture. The tubes containing the mixture were exposed to UV, and digital images were acquired by the GelDoc System (BioRad Laboratories, Milan, Italy).

### PCR assays

In order to confirm the results gathered by LAMP, and to compare the sensitivity of LAMP and traditional PCR, a COI-targeting PCR assays was performed according to the protocol by Casiraghi et al. [29], by using the primers COIintF and COIintR, which are expected to return a 689-pb amplicon from many nematoda species, including those considered in this study.

The products were analyzed in a 1.5% agarose gel and visualized after dying with ethidium bromide 0.5 μg/mL. Images were digitalized by the mean of a Gel Doc EZ system (BioRad Laboratories, Milan, Italy).

### Specificity tests

A preliminary specificity assay was performed *in silico* by comparing the primer sequences with the full or partial COI sequences listed in Table 2. The comparison was performed by BLAST [30]. The nucleotide sequences of *DiR*F1 and *DiR*F2c (which constituted *DiR*FIP), and of *DiR*B1 and *DiR*B2c (*DiR*BIP) were split and individually checked. Matches were considered significant when a portion at least corresponding to 95% of the sequence of the checked oligonucleotide was at least 90% identical to the target sequence, without mismatches in the last three bases of the 3' termini.

**Table 2:** Parasites tested for specificity assays and their respective results.

Parasite	Identification number	*In silico* test						*In vitro* test
	(GenBank/IZSPB accession number)	*DiR*F1	*DiR*F2	*DiR*F3	*DiR*B1	*DiR*B2	*DiR*B3	
*Acanthocheiloma reconditum*	IZSPB03004^‡^	NP						neg
*Acanthocheilonema* sp.^‡^	KX032518*	+	-	-	-	-	-	neg
*Angiostrongylus vasorum*	IZSPB01012^‡^	NP						neg
*Brugia malayi*	AF538716	+	-	-	-	-	+	NP
*Brugia timori*	LK926830	+	-	+	-	+	-	NP
*Brugia* sp.^§^	-	NP						neg
*Cercopithifilaria shohoi*	AM749251	+	-	+	-	-	-	NP
*Cercopithifilaria* sp. ^†^	-	NP						neg
*Dirofilaria immitis*	AJ537512	+	+	+	-	-	-	NP
*Dirofilaria immitis*^‡^	-	NP						neg
*Eleaphora elaphi*	LL712680	+	-	-	-	+	-	NP
*Litomosoides brasiliensis*	AJ544867	-	-	+	.	+	-	NP
*Loa loa*	HQ186250	+	-	-	-	-	-	NP
*Loa loa*^§^	-	NP						neg
*Mansonella perstans*^§^	KU215907*	-	-	-	-	+	-	neg
*Onchocerca dewittei*	AB518690	+	-	+	-	+	+	NP
*Onchocerca eberhardi*	AM749268	-	-	+	+	-	+	NP
*Onchocerca gutturosa*	AJ271617	+	-	+	-	+	+	NP
*Onchocerca jakutensis*	KT001213	+	-	-	-	+	-	NP
*Onchocerca volvulus*	KT599912	+	-	-	-	-	+	NP
*Setaria labiatopapillosa*	AJ544872	+	-	+	-	-	+	NP
*Spirocerca lupi*	KC305876	-	+	+	-	-	-	NP
*Spirocerca lupi*^‡^	IZSPB1704	NP						neg
*Spirocerca* sp.	KJ605489	-	+	+	-	-	+	NP
*Thelazia callipaeda*	JX069968	+	+	+	-	-	-	NP
*Wuchereria bancrofti*	JQ316200	+	+	-	-	+	+	NP
*Wuchereria bancrofti*^§^	-	NP						neg

NP: Not performed; neg: negative to in vitro test;

^+^: oligonucleotide matching a corresponding sequence onto the target COI;

^-^: oligonucleotide not matching any sequence onto the target COI.

* Nucleotide sequence determined in this study;

The specimen tested in vitro were from:

^‡^ the collection of the Experimental Zooprophylactic Institute of Apulia and Basilicata;

^§^ the collection of the Tropical Diseases Unit, “Sacro Cuore” Hospital, Negrar, Verona;

^†^ the Prof. Domenico Otranto’s collection

*In vitro* specificity assays were performed by using DNA from previously identified nematode species such as *Dirofilaria (D*.*) immitis*, *Acanthocheilonema (Ac*.*) reconditum*, *Angiostrongylus (An*.*) vasorum*, *Brugia* sp., *Loa (L*.*) loa*, *Wuchereria (W*.*) bancrofti*, *Mansonella (M*.*) perstans* and *Cercopithifilaria* sp.. Positive controls were carried out with genomic DNA from *D*. *repens*.

In order to check the reliability of the test with more complex starting samples, it was also performed by using DNA from canine blood and mosquitoes.

Specifically, 40 canine blood samples were used. They were collected from the cephalic vein of dogs hosted in a dog kennel and previously checked by the qPCR method described by Czajka *et al*. [31], designed to reveal the presence of Onchocercidae. Out of the forty blood samples, twelve (12/30) were positive.

Arthropod samples consisted of 46 pools of Culicidae mosquitoes belonging to the collection of IZSPB and previously collected in Southern Italy during a research project (R.C. IZSPB 005/2010) funded by Italian Minister of Health. The samples were also screened for the presence of filarial nematodes, and 6 of them were found positive [32]. An additional negative control, consisting of a pool of 10 *Dirofilaria*-free *Aedes albopictus* mosquitoes from IZSPB laboratory colony, was also included.

All samples (genomic DNA, blood and arthropod samples) were tested by performing both the ReT-LAMP and PI-LAMP protocols.

In order to definitively confirm the presence or absence of *D*. *repens*, the blood and mosquitoes samples were also tested by using the COI-targeting PCR [29]. The yielded amplicons were sequenced by the BigDye Terminator v3.1 (Applied Biosystems, Milan, Italy), and the nucleotide sequences were compared with those from GenBank by BLAST.

### Sensitivity test

A COI-targeting PCR [29] was performed by using DNA from *D*. *repens* larvae as template. Larvae were part of the collection of IZSPB, and they were previously isolated from the blood of an infected dog. The yielded 689-bp amplicon, which included the LAMP-targeted region, was purified by the mean of the QIAquick PCR purification kit, cloned in pGEM T-easy vector (Promega, Milan, Italy) according to the manufacturer's instructions, and then transformed in *Escherichia coli* MACH1.

The recombinant plasmid was extracted from a positive clone by using the PureLink HiPure plasmid miniprep kit (Thermo Scientific, Milan, Italy). Following quantification by the mean of a UV spectrophotometer, ten-fold serial dilution of plasmid DNA were prepared, from an initial concentration of 30 ng/μL, corresponding to about 8,5x10^9^ copies/μL, up to a concentration of 30 ag/μL (8–9 copies/μL).

Five μL of each dilution was used as template for ReT-LAMP, PI-LAMP and PCR assays.

### Ethical clearance

The blood samples from stray dogs (without any known owner) were collected by professional veterinarians without causing injury or any kind of consequence to the animals. The blood collection was performed as a routine, according to the local rules, upon admittance to the dog kennel, to assess or exclude the presence of infectious diseases. No animal was sacrificed or euthanatized for the aims of this study.

## Results

### Specificity tests

When tested *in silico*, all but one tested genes harbored potential annealing regions for no more than three out of the six oligonucleotides which constituted the LAMP primer set. In no cases the sequences of *DiR*F2c and *DiR*B2c, which prime the initial amplification step, matched together within the COI of the same species (Table 2). The only exception was represented by the COI of *W*. *bancrofti*, which was found to harbor the complementary motif of four oligonucleotides, including *DiR*F2c and *DiR*B2c.

**Fig 2:**
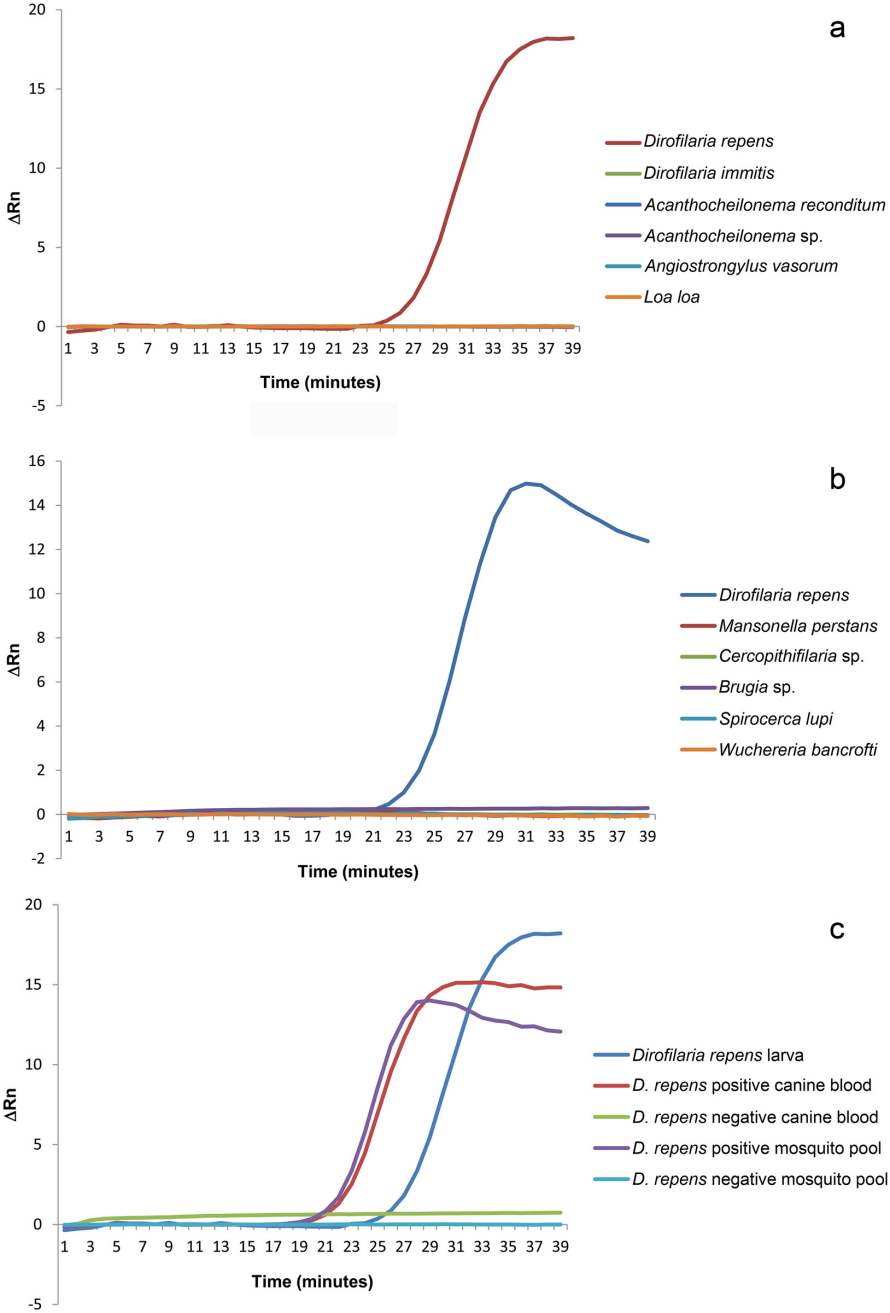
Specificity of the ReT-LAMP. The curves represent the amplification signals expressed as ΔRn, after subtraction of the ROX reference dye fluorescence. a) and b) Amplification curves from ReT-LAMP carried out with DNA from larvae of previously identified species. The curves from species different from *D*. *repens* are overlapped because of the lack of an appreciable signal. c) Amplification curves from DNA extracted from biological samples (mosquitoes and canine blood).

**Fig 3:**
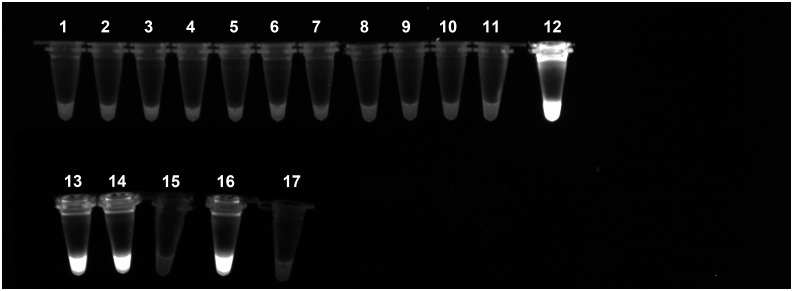
Specificity of the PI-LAMP. The micro-tubes containing the reaction mixture with propidium iodide have been exposed to UV after incubation. The positive reactions that returned an amplification product are evidenced by a bright fluorescence. The reaction have been performed by using purified DNA from larvae of 1) *Acanthocheilonema reconditum*; 2) *Acanthocheilonema* sp.; 3)*Angiostrongylus vasorum*; 4) *Brugia* sp.; 5) *Cercopithifilaria* sp.; 6) *Dirofilaria immitis*; 7) *Loa*; 8) *Mansonella perstans*; 9) *Spirocerca lupi*; 10) *Wuchereria bancrofti*; 12) and 13) *Dirofilaria repens*; 14) *D*. *repens* positive mosquito pool; 15) *D*. *repens* negative mosquito pool; 16) *D*. *repens* positive canine blood; 17) *D*. *repens* negative canine blood. The sample 11 is a negative sample with water instead of DNA.

However, when it was tested *in vitro*, no amplification was showed. Equally, no amplification was registered when DNAs from samples of *D*. *immits*, *Ac*. *reconditum*, *An*. *vasorum*, *Brugia* sp., *L*. *loa*, *M*. *perstans* or *Cercopithifilaria sp*. were used as template (able 2). On the contrary, all the ReT:LAMP reactions with DNA of *D*. *repens* returned the expected amplification curve (Fig 2a and 2b), and all the PI-LAMP mixtures were fluorescent (Fig 3).

Similarly, when the LAMP assays were performed with DNA from blood and mosquitoes samples, all the samples that were previously found positive to Onchocercidae resulted positive to ReT-(Fig 2c) and PI-LAMP (Fig 3), while no amplification was observed from the other samples.

The positive samples, when tested by the mean of the COI-targeting PCR, returned the expected 689-bp amplicon. The nucleotide sequences of amplicons were 98–100% identical to those in GenBank from *D*. *repens*.

### Sensitivity test

**Fig 4:**
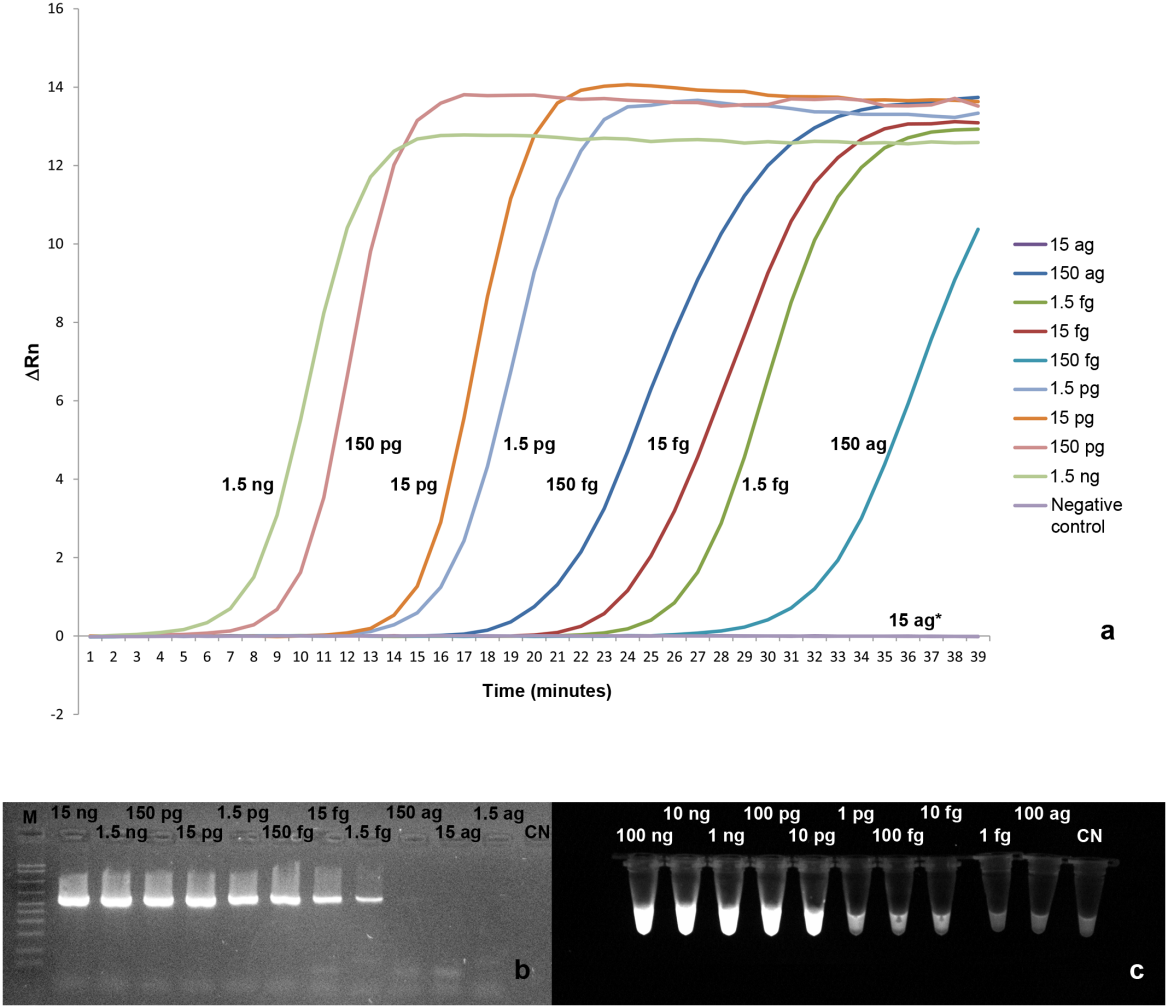
Results of the sensitivity assay. a) ReT-LAMP. The curves represent the ΔRn after subtraction of the ROX reference dye fluorescence. The amount of *D*. *repens* DNA for each reaction is indicated near the respective curve. *The curves of the reaction with 15 ag of DNA and of negative control are overlapping. b) PCR. M: AmpliSize Molecular Ruler, 50–2,000 bp Ladder (BioRad Laboratories, Milan, Italy). c) PI-LAMP. The bright fluorescence indicates the positivity of the reaction.

The minimum amount of template necessary to obtain an amplification profile by the ReT LAMP was 0.15 fg, corresponding to about 50 copies of target (Fig 4a), while an amplicon was yielded by PCR (Fig 4b) from a minimum starting amount of 15 fg (about 5,000 copies of target).

Conversely, the PI-LAMP showed a detection limit of 10 fg (about 3,000 copies of target (Fig 4c).

## Discussion

The broad diffusion of the dirofilariasis due to *D*. *repens* stresses the needing of suitable and affordable diagnostic tools, which might promptly detect the parasite in hosts or in vectors.

The presented results showed that LAMP may be a suitable approach for the laboratory confirmation of the *D*. *repens* infection. Currently, the diagnosis of dirofilariasis, due to *D*. *repens* or *D*. *immitis*, relies on the microscopic and morphological identification of the parasite, usually microfilariae isolated from the blood of infected hosts [33]. Other methods are based on serological screenings [34–35] or on the detection of DNA of the nematode. In particular, PCR-based strategies are currently described to amplify *D*. *repens* DNA from mammals [36–40] or mosquitoes samples [11, 31, 41]. Among the diagnostic molecular methods, the LAMP is a promising system, and nowadays, it is applied for the diagnosis of fungal, viral, bacterial and parasitic infections [28, 42–43], including zoonotic filarial nematodes, such as the canine heartworm *D*. *immitis*, [44], *W*. *bancrofti* [45], the human lymphatic filarial nematodes *Brugia malayi* and *B*. *timori* [46]. The here described assay represents the first LAMP protocol for the detection of *D*. *repens* DNA. It results highly sensitive, as it returns a detectable amplification product from 0.15 fg of template, corresponding to about 50 copies of target, when performed as ReT-LAMP. While lower than ReT-LAMP, the sensitivity of PI-LAMP remains high, closely consistent with the traditional PCR. The specificity remains high, as well. In fact, no false positive result was obtained, while all samples that resulted positive by bother methods were also ReT-and PI-LAMP positive, thus confirming the cogency of the method.

Both versions of LAMP can be performed in about 40 minutes, with a faster outcome than PCR, which takes about 2 hours. Additionally, the here described LAMP protocols are also faster than the previously described qPCR assays [31, 41], which takes about 100–150 minutes. Furthermore, the latter were designed and developed to target a wider range of organisms (both *D*. *repens* and *D*. *immitis* [41], or a large group of filarial nematodes [31]). Therefore, the species identification must rely on the analysis of the melting curve or the nucleotide sequence, and this make those methods more demanding in terms of time and work.

Furthermore, the amplification may be immediately visualized, without the need for an agarose gel electrophoresis. Finally, the results can be unequivocally interpreted, as the amplification curve (for ReT-LAMP) and fluorescence (for PI-LAMP) were clearly visible from positive samples, while no aspecific signal was observed from negative samples. In addition, the here described LAMP assays appear to be effective on different matrices: the reactions carried out from blood and mosquito specimens returned clear amplification signals when the parasite was present in the sample, while no kind of signal was found from those negative.

In the light if those consideration, the described method represents, to our knowledge, the first LAMP-based tool to detect *D*. *repens* directly from biological samples.

This makes the assay effective for the detection of *D*. *repens* in hosts with microfilaraemia, and it may reliably support the differential diagnosis with *D*. *immitis*, *Ac*. *reconditum* or *Mansonella* spp.. Therefore, a potential application of the method may be the screening of traveling live animals, which are often responsible for the introduction of the parasite in unaffected areas. Furthermore, the PI-LAMP, being a rapid and cost-effective assay, could be an useful and ancillary tool for screening a large number of culicid mosquitoes and to assess their positivity for *D*. *repens* during entomological surveys in endemic, or even in non-endemic areas. Finally, the PI-LAMP protocol can also be performed with very simple equipment, such as a heating device instead of the more expensive real time PCR instrument. This version has been found to be slightly less sensible if compared with the ReT-LAMP protocol, but it did not show any impairment in the specificity of the assay. This possibility could make the method suitable for application in field, especially in developing Countries, where expensive equipment and specialized personnel may often lack.

## Supporting Information

S1 Text Brief description of general principles and mechanism of LAMP reaction. (DOC)
