# Supplementary material for: Development and Application of a Loop-Mediated Isothermal Amplification (LAMP) Approach for the Rapid Detection of Dirofilaria repens from Biological Samples
Source: PLoS Negl Trop Dis. 2016 Jun 24;10(6):e0004789. doi: 10.1371/journal.pntd.0004789 (PMC4920375; doi:10.1371/journal.pntd.0004789)
Supplement: S1 Text — (DOC) [file pntd.0004789.s001.doc]

The technique consists in a targeted DNA amplification catalyzed by a DNA polymerase with strand displacement activity, usually the *Bst* DNA polymerase. The reaction is primed by two pairs of oligonucleotides (Fig. 1), termed inner and outer primers (FIP/BIP and F3/B3, respectively). Although FIP and BIP are a single DNA molecule, they may be functionally split in two segments, F1c and F2, and B2 and B1c, respectively. F2 and B2 anneal to their respective targets in the template, and they prime the *Bst* DNA polymerase catalyzed synthesis of the complementary strand. The neosynthesized strands are then displaced from the template by the synthesis of another strand, primed by F3 and B3, which anneal upstream from F2 and B2, respectively. Hereafter, the process will be only described for one of the two strands, considering that the other undergoes the same reaction chain, in the opposite sense.

Downstream from F2, the neosynthesized, displaced strand harbors F1, the annealing target of F1c, B2c which is recognized by B2, and B3c, which may be hybridized by B3.

On the next stage, F1c self-anneals to F1, and, in the opposite direction, B2 anneals with B2c and B3 with B3c. In this way, two concurrent synthesis processes take place: the first is primed by F1c, the second by B2, and the third by B3. B1c does not prime any DNA synthesis as it terminally exposes the 5' end. Since the strand from B3 displaces the one starting from B2, this step will give two distinct products: the first is a double stranded DNA molecule starting from B3 and ending to F1, and the second owns a typical dumbbell form. The latter is the starting point for the following amplification steps. The self-hybridization of F1, with the free 3' end, provides a priming site for the synthesis of a complementary strand, which will displace the 5' end annealed on the other terminus. This results in the production of a stem-loop structure, in which the stem is extended from F1 (annealed to F1c) to B1 (annealed to B1c). The single stranded loop includes F2c. This will act as a recognition site for F2, linked to F1c in the FIP oligonucleotide, which, in turn, will prime the new strand displacement DNA synthesis, in which the stem-loop molecule will act as a template. The synthesized DNA strand harbors the hybridization site for the B2 portion of the BIP oligonucleotide, which will prime the strand displacement DNA synthesis of the complementary strand. This results in an amplification cycle. The final products is a mixture of concatamers with stem and loop structure, variable in dimension, according to the number of amplification steps which a molecule underwent to.
